# Relationship Between Health-Related Quality of Life and Exercise Tolerance Improvement in Remote Cardiac Rehabilitation: Sub-Analysis of RecRCR Study

**DOI:** 10.3390/jcm14103265

**Published:** 2025-05-08

**Authors:** Mai Shimbo, Eisuke Amiya, Takahiro Jimba, Hidetaka Itoh, Koichi Narita, Masanobu Taya, Toshiaki Kadokami, Takanori Yasu, Hideki Oka, Masakazu Sogawa, Hiroyoshi Yokoi, Kazuo Mizutani, Shin-ichiro Miura, Tatsuo Tokeshi, Ayumi Date, Takahisa Noma, Daisuke Kutsuzawa, Soichiro Usui, Shigeo Sugawara, Masanori Kanazawa, Hisakuni Sekino, Miho Nishitani Yokoyama, Takahiro Okumura, Yusuke Ugata, Shinichiro Fujishima, Kagami Hirabayashi, Yuta Ishizaki, Koichiro Kuwahara, Yuko Kaji, Hiroki Shimizu, Teruyuki Koyama, Hitoshi Adachi, Yoko Kurumatani, Ryoji Taniguchi, Katsuhiko Ohori, Hirokazu Shiraishi, Takashi Hasegawa, Shigeru Makita, Issei Komuro, Norihiko Takeda, Yutaka Kimura, RecRCR Registry Investigators

**Affiliations:** 1 Department of Cardiovascular Medicine, Graduate School of Medicine, The University of Tokyo, Tokyo 113-8654, Japan; shimbomy@gmail.com (M.S.);; 2 Department of Computational Diagnostic Radiology and Preventive Medicine, The University of Tokyo, Tokyo 113-8655, Japan; 3 Saiseikai Futsukaichi Hospital, Fukuoka 818-8516, Japan; 4 Department of Cardiovascular Medicine and Nephrology, Dokkyo Medical University Nikko Medical Center, Tochigi 321-1298, Japan; 5 Nijigaoka Hospital, Nagasaki 851-0116, Japan; 6 Moriyama Memorial Hospital, Tokyo 134-0081, Japan; 7 Fukuoka Sanno Hospital, Fukuoka 814-0001, Japan; 8 Rokko Island Konan Hospital, Hyogo 658-0032, Japan; 9 Department of Cardiology, School of Medicine, Fukuoka University, Fukuoka 814-0180, Japan; 10 Shonan Hospital, Okinawa 904-0034, Japan; 11 Asahikawa Medical University Hospital, Hokkaido 078-8510, Japan; 12 Department of Cardiorenal and Cerebrovascular Medicine, Faculty of Medicine, Kagawa University, Kagawa 761-0793, Japan; 13 Department of Cardiology, Pulmonology, and Nephrology, School of Medicine, Yamagata University, Yamagata 990-9585, Japan; 14 Department of Cardiovascular Medicine, School of Medical Science, Kanazawa University, Kanazawa City 920-8641, Japan; 15 Nihonkai General Hospital, Yamagata 998-8501, Japan; 16 Department of Cardiology, Iwate Prefectural Central Hospital, Morioka 020-0066, Japan; 17 Department of Cardiac Surgery, Association of Healthcare Corporation Kyufukukai Sekino Hospital, Tokyo 107-0052, Japan; 18 Department of Cardiovascular Biology and Medicine, Graduate School of Medicine, Juntendo University, Tokyo 113-8421, Japan; 19 Department of Cardiology, Graduate School of Medicine, Nagoya University, Nagoya 466-8550, Japan; 20 Jichi Medical University Saitama Medical Center, Saitama 330-8503, Japan; 21 Cardiovascular Center, Steel Memorial Yawata Hospital, Fukuoka 805-8508, Japan; 22 Department of Cardiology, Tomakomai City Hospital, Tomakomai 053-8567, Japan; 23 School of Medicine, Kurume University, Fukuoka 830-0011, Japan; 24 Department of Cardiovascular Medicine, Shinshu University School of Medicine, Nagano 390-8621, Japan; 25 Department of Nursing, Hiraka General Hospital, Akita 010-0001, Japan; 26 Department of Cardiology, Konan Medical Center, Kobe 658-0064, Japan; 27 Kameda Medical Center, Chiba 296-8602, Japan; 28 Gunma Prefectural Cardiovascular Center, Maebashi 371-0004, Japan; 29 Kofu Kyoritsu Hospital, Yamanashi 400-0805, Japan; 30 Hyogo Prefectural Amagasaki General Medical Center, Amagasaki 660-8550, Japan; 31 Department of Cardiology, Hokkaido Cardiovascular Hospital, Sapporo 060-8638, Japan; 32 Department of Cardiovascular Medicine, Graduate School of Medical Science, Kyoto Prefectural University of Medicine, Kyoto 602-8566, Japan; 33 Japan Telemedicine Society, Japan; 34 Kawaguchi Cupola Rehabilitation Hospital/Saitama Medical University International Medical Center, Saitama 350-1298, Japan; 35 International University of Health and Welfare, Tokyo 324-8501, Japan; 36 Department of Frontier Cardiovascular Science, Graduate School of Medicine, The University of Tokyo, Tokyo 113-0033, Japan; 37 Kansai Medical University, Osaka 573-1191, Japan

**Keywords:** remote cardiac rehabilitation, quality of life, exercise tolerance, real-time telemonitoring, SF-8

## Abstract

**Background:** Remote cardiac rehabilitation (RCR) is emerging alternative to outpatient rehabilitation. However, evidence related to its effect on health-related quality of life (HRQOL) is limited. **Methods:** This is a sub-analysis of the RecRCR study, a multi-center, nonrandomized trial evaluating the efficacy and safety of RCR with real-time telemonitoring in patients with CVD, after discharge. The Short-Form Health Survey-8 was employed to evaluate the HRQOL before and 2–3 months after RCR. Based on the improvement of exercise tolerance, the patients were divided into I group (>10% improvement) and NI group (≤10% improvement). **Results:** Of 57 patients who completed RCR, 31 patients were included for analysis of HRQOL, including 15 (I group) and 16 patients (NI group). The physical (PCS) (45.5 ± 8.0 to 52.5 ± 4.0, *p* < 0.001) and mental (MCS) component scores (47.5 ± 7.9 to 51.0 ± 5.6, *p* = 0.005) improved significantly in all populations following RCR. The PCS improved significantly in the I and NI groups, respectively. By contrast, only in the I group, the MCS improved. However, the change in PCS or MCS was not significantly different between the two groups. The increases of MCS significantly associated with days from admission to the beginning of RCR (rs = −0.51, *p* = 0.007). **Conclusions:** In multifaced contents of HRQOL, the scores in PCS or MCS changed differently from the change in exercise capacity.

## 1. Introduction

Considering that most patients with cardiovascular diseases (CVDs) experience functional disabilities that impair their quality of life (QOL) and increase the risk of mortality, it is important to consider their QOL [1,2]. Cardiac rehabilitation enhances exercise capacity and health-related quality of life (HRQOL) in patients with CVD [3,4]. However, poor adherence to the rehabilitation program is a major issue that can significantly hinder the achievement of the anticipated outcomes [5].

Remote cardiac rehabilitation (RCR) is an emerging alternative to outpatient rehabilitation [6,7]. Anderson et al. reported that home- and center-based forms of cardiac rehabilitation offer equivalent efficacy in improving clinical- and health-related quality of life outcomes in patients with CVD [8]. We previously reported the efficacy and safety of RCR with real-time monitoring, using a bidirectional communication tool on exercise tolerance in the recovery phase, which was comparative to center-based outpatient cardiac rehabilitation [9]. However, there is limited evidence on the effect of RCR with real-time telemonitoring on HRQOL during the recovery phase of patients discharged after in-hospital treatment. Furthermore, little had been known about the relationship between HRQOL and exercise capacity during RCR.

This study aimed to investigate the relationship between the changes in HRQOL and exercise capacity in CVD patients receiving RCR.

## 2. Materials and Methods

### 2.1. Study Patients

This is a sub-analysis of the RecRCR study, a multi-center, nonrandomized trial evaluating the efficacy and safety of RCR with real-time telemonitoring among patients with CVD who were indicated for CR in the recovery phase. Details regarding the design and primary trial results of the RecRCR study have been published previously [9,10]. In brief, the study recruited 57 participants as the RCR group from 14 January 2021, to 31 March 2021. The study was conducted in accordance with ethical guidelines and the Declaration of Helsinki. This clinical trial was registered in the University Hospital Medical Information Network—Clinical Trials Registry (UMIN–CTR: UMIN000042942). The protocol was approved by the institutional review board of Tokyo University Hospital (2020305NI), and all the study participants provided written informed consent for participation.

In the present sub-analysis, the patients were divided into two groups based on the improvement of exercise tolerance after 2–3 months of RCR: I group (>10% improvement of peak oxygen uptake measured by cardiopulmonary exercise testing (CPET) or distance by 6-min walk test) and NI group (no improvement in exercise tolerance) [11].

### 2.2. Intervention

The details of RCR with real-time telemonitoring, as implemented in the RecRCR study, have been reported previously [9,10]. Patients in the RCR group used devices including calibrated ergometers and tablets for face-to-face communication during exercise and for receiving e-learning guidance necessary for undergoing RCR after discharge from the hospital. The patients underwent aerobic exercise sessions using an ergometer while being monitored by medical professionals via real-time video conversation using a provided device. An instructor monitored the patients’ status and vital signs by interactive video tools during exercise. Meanwhile, video-based learning was implemented using an attached tablet during RCR. The session was held three times a week, with each session of 30–40 min. The e-learning content included information on CVD risks, nutrition, and lifestyle modifications for disease control.

### 2.3. Health-Related QOL Assessment

HRQOL was assessed using the Short-Form Health Survey-8 (SF-8) before and after 2–3 months of RCR. The validity and reliability of the Japanese version of SF-8 have been previously documented [12]. In the context of SF-8, higher scores represent better QOL. The physical health component summary score (PCS), mental health component summary score (MCS) and eight subscales (e.g., physical functioning, role physical, bodily pain, general health, vitality, social functioning, role emotional, and mental health), were assessed.

### 2.4. Statistical Analysis

Continuous variables are presented as the mean (standard deviation) and median (quadrant). These variables were compared using the Student’s *T*-test for normally distributed data and the Mann–Whitney U test for data that do not follow a normal distribution. To check the normality of each parameter, we used Shapiro-Wilk test. Categorical variables are expressed as frequencies and percentages and were compared using chi-square tests. Categorical and continuous variables were compared between patients who experienced an improvement in exercise tolerance following RCR and those who did not. Baseline and follow-up data were compared using the paired T-test for normally distributed data and Wilcoxon signed-rank sum test for data that do not follow a normal distribution. The correlation coefficients between changes in PCS and MCS and variables (e.g., laboratory data or exercise tolerance at baseline), and the number of times and days of RCR and days from admission to the beginning of RCR were determined by Spearman’s rank analyses. Changes in exercise capacity, PCS, and MCS at baseline and follow-up between patients in the I and NI groups were compared using analysis of covariance (ANCOVA). All statistical analyses were performed using SPSS software v22 (IBM Inc., Armonk, NY, USA), and statistical significance was considered at *p* < 0.05.

## 3. Results

### 3.1. Patient Characteristics

**Figure 1:**
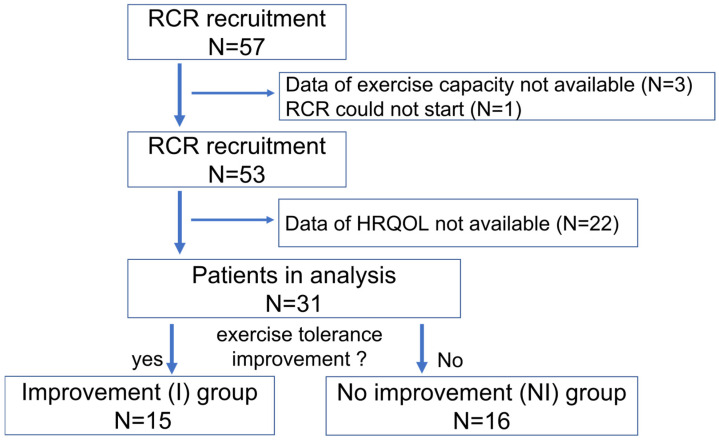
Flow chart of the participants. Abbreviations: RCR; remote cardiac rehabilitation, HRQOL; health related quality of life.

Fifty-seven patients were enrolled in the RCR group between 14 January 2021, and 31 March 2021. Of the 57 patients, one withdrew from the clinical trial, and those who lacked data on exercise capacity and HRQOL at baseline and follow-up were excluded. Finally, this study included 31 patients with CVD (Figure 1), among whom 15 (48.4%) and 16 (51.6%) were assigned to the I and NI group, respectively. The patient characteristics are shown in Table 1. The mean age of participants in the I and NI groups was 61.8 and 64.6 years (*p* = 0.582), respectively. Both groups predominately consisted of men (80.0% vs. 87.5%, *p* = 0.654). The most common indication for administering CR was ischemic heart disease (60.0%), followed by heart failure (33.3%) in the I group. There were no significant differences in laboratory data and medications between the two groups. In the two groups, peak oxygen uptake and 6MWT distance were 14.8 ± 4.7 mL/kg/min and 17.2 ± 5.1 mL/kg/min, respectively, and 376.3 ± 102.7 and 372.0 ± 68.5 m, respectively (*p* = 0.269 and 0.935, respectively). Further, there were no differences in the number of times RCR was conducted, the duration of days of RCR (in days), and the days from hospitalization to RCR between the two groups.

### 3.2. HRQOL Measured by SF-8

**Figure 2:**
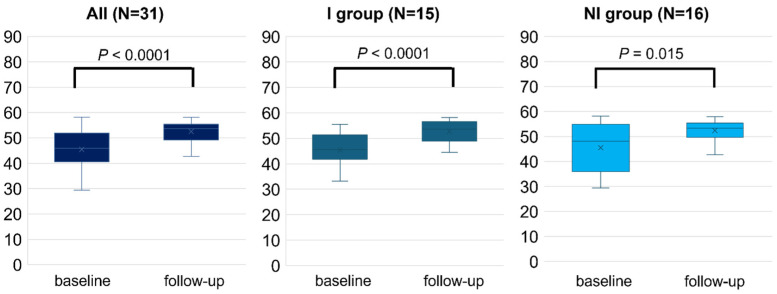
Physical health component score (PCS) measured by SF-8 before (baseline) and after 2–3 months (follow-up) of RCR. The comparison of PCS between baseline and follow-up was performed in all populations, I and NI groups. Length of the box is the difference between 75th and 25th percentiles and a line is drawn on the median.

**Figure 3:**
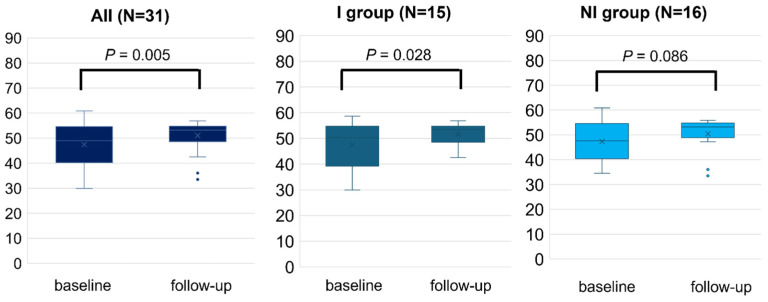
Mental health component score (MCS) measured by SF-8 before (baseline) and after 2–3 months (follow-up) of RCR. The comparison of MCS between baseline and follow-up was performed in all populations, I and NI groups. Length of the box is the difference between 75th and 25th percentiles and a line is drawn on the median.

Overall, the study patients experienced a significant change in all subscales (*p* < 0.05), including the PCS (45.5 ± 8.0 to 52.5 ± 4.0, *p* < 0.001) and MCS (47.5 ± 7.9 to 51.0 ± 5.6, *p* = 0.005), following RCR. After dividing into two groups by the improvement of exercise capacity, both groups exhibited significant changes in PCS after 2–3 months of RCR (I, 45.3 ± 6.4 to 52.7 ± 3.9, *p* < 0.0001; and NI, 45.5 ± 9.5 to 52.4 ± 4.1, *p* = 0.015, Figure 2). By contrast, the change in MCS scores achieved statistically significance in the I group (47.5 ± 8.5 to 51.7 ± 4.3, *p* = 0.028), but did not in the NI group (47.3 ± 7.5 to 50.4 ± 6.6, *p* = 0.086) (Figure 3). The I group demonstrated a significant change in all eight subscales, except for bodily pain, as indicated by each specific score. Conversely, only four subscales including physical functioning, general health, vitality, and social functioning changed in the NI group (Table 2). Further, the difference in the change of exercise capacity, exemplified by peak oxygen uptake or 6MWT distance, was significant in two groups (*p* < 0.001), while the change in PCS or MCS was not significantly different between the two groups by ANCOVA (PCS; *p* = 0.80, MCS; *p* = 0.51).

### 3.3. Correlations Between Changes in PCS, MCS and Various Parameters

According to the determining factors of the change of HRQOL scores by RCR, the increase of PCS did not correlate with any variables including demographic data or days of RCR. On the other hand, the increases of MCS significantly associated with days from admission to the beginning of RCR (r_s_ = −0.51, *p* = 0.007) (Table 3). Neither of the changes in PCS and MCS were correlated with the change in exercise capacity.

## 4. Discussion

The major findings of the study were: (1) the scores in HRQOL changed after 2–3 months of RCR via real-time telemonitoring following discharge. (2) PCS changed significantly regardless of improvement in exercise tolerance in both the groups while the improvement of MCS was blunted in the NI group. There was a dissociation between the changes in PCS or MCS and exercise capacity. (3) The increases of MCS significantly associated with days from admission to the beginning of RCR.

### 4.1. Change in HRQOL During RCR

Center-based CR has been established as an effective intervention for improving HRQOL [13]. The consistent efficacy of RCR in enhancing HRQOL has been demonstrated in some reports [14,15]. Taylor et al. showed that exercise-based CR, including the home-based CR, is effective in improving the HRQOL of patients with heart failure [15]. Peng et al. reported that implementing RCR measures, including telehealth exercise training, education, and consultations by cardiac nurses, significantly improved HRQOL in patients with heart failure presenting clinically stable conditions [14]. As described above, it is possible to expect RCR to have an effect on improving HRQOL. However, RCR methods vary greatly, such as the presence or absence of real-time monitoring, face-to-face communication, and simultaneity with other participants, and each element is likely to have a different effect on QOL. In particular, providing comprehensive disease management, which includes exercise training based on exercise capacity, psychological support, and the learning of self-management skills, has much importance during the recovery phase [13]. Thus, it is crucial to provide an appropriate form of RCR in patients with CVD at that stage.

The interesting finding of our study is that physical QOL (PCS) improved significantly, despite the fact that the value of peak VO_2_ did not significantly increase. Mansilla-Chacón M et al. reported that the self-reported measures of physical activity of patients after myocardial infarction were statistically improved based on the dimensions of the Myocardial Infarction Dimensional Assessment Scale in supervised CR programs [16]. Moreover, the improvement of HRQOL was related to the enhancement in exercise tolerance in both center-based and home-based CR [17,18]. Conceivably, other effects than those derived from exercise training, such as face-to-face communication, counseling by professional staff, and e-learning-based patient education, could be expected in our study of RCR [19,20,21]. Improvement of HRQOL was reported to be associated with the improvement of clinical events [22,23], which could allow us to consider HRQOL as another crucial outcome in CR. Nevertheless, we could not demonstrate the efficacy of RCR on HRQOL because of the lack of control and sufficient sample size. We should further verify it by more robust study with comparative control and larger sample size.

### 4.2. Relationship Between HRQOL Score and Exercise Capacity During RCR

There had been several reports about the relationship between exercise capacity and HRQOL. Several reports demonstrated dissociations between HRQOL and exercise capacity [24,25]. On the other hand, some rehabilitation-related reports have shown that HRQOL improves in parallel with improvements in exercise tolerance [26,27].

Our results suggest that some HRQOL scores will change in correlation with changes in exercise capacity, while overall HRQOL values changed in a manner different from exercise tolerance. Indeed, while PCS was thought to be more likely to be linked, MCS was unexpectedly linked, and PCS virtually tended to improve even without improvement in exercise capacity. These results suggest that the measures to promote specific improvement in MCS was insufficient in the current study. Although patient education and face-to-face communication were indeed carried out, this was insufficient to improve MCS independently of exercise tolerance, suggesting that specific support from psychological experts should be considered.

### 4.3. Limitaions

This study had several limitations. First, the effect on HRQOL was evaluated for a short time period. Therefore, the long-term effects of the clinical events are unconfirmed. The limited number of patients included in the study may have increased the likelihood of type 2 errors. The absence of statistically significant differences between two groups for HRQOL outcomes might be derived from low statistical power due to small sample size. We should verify the findings of this study by future studies with larger sample size including control comparisons. Second, the evaluation method for exercise capacity could not be standardized in this study because some facilities were unable to conduct CPET due to the coronavirus pandemic 2019, so as providing center-based CR that resulted in the lack of data of HRQOL of control group. However, there is a certain degree of correlation between peak VO2 in CPET and 6-min walking distance [28]. Moreover, the absence of a simultaneously recruited control is one of the major limitations in this study. These limitations rendered the results of this study inconclusive. In the future, it is imperative to conduct a further study to confirm the efficacy of RCR. We conducted interventions that included expert consultation, e-learning, and exercise therapy. However, we could not perform counseling from a mental support professional. It is also expected that the psychological effects can be further increased by adding the specialized support of a psychological counselor. Furthermore, telemedicine, including e-learning, can be used not only for RCR, but also in the following clinical situations. (Online consultations by experts for patients living far away, specialized consultations by psychological counselors in situations where the mental condition is deteriorating, etc.) [29] By establishing a flow for consultations by experts using remote technology, it is possible to continue interventions that can achieve clinical effects even if they are separated from RCR. Last, because the threshold of 10% in the improvement of exercise tolerance had not been sufficiently justified, we performed sensitivity analysis using different thresholds in the improvement of exercise tolerance (Supplementary Materials File S1). The results were similar, which would suggest the validity of the result in more robust way. In addition, the RCR equipment was purchased using research funds independent of device manufactures.

## 5. Conclusions

The incorporation of RCR interventions with real-time telemonitoring might improve the scores in HRQOL. The scores in PCS or MCS changed differently from the change in exercise capacity during RCR.

## Figures and Tables

**Table 1:** Baseline characteristics of the study population. The data are presented as “n (%)” or “mean (standard deviation)” or “median (quadrant) ”.

	I Group N = 15	NI Group N = 16	*p*-Value
Age (years)	61.8 ± 13.6	64.6 ± 14.7	0.582
Gender male (%)	12 (80.0%)	14 (87.5%)	0.654
BMI (kg/m^2^)	24.5 ± 5.2	23.8 ± 3.3	0.673
**Indication for CR (%)**			0.300
Ischemic heart disease, n (%)	9 (60.0%)	6 (37.5%)	
Heart failure, n (%)	5 (33.3%)	6 (37.5%)	
Post cardiac surgery, n (%)	1 (6.7%)	1 (6.3%)	
Others, n (%)	0 (0.0%)	0 (0.0%)	
**Risk factors**			
Hypertension, n (%)	8 (53.3%)	9 (56.3%)	0.870
Diabetes, n (%)	4 (26.7%)	3 (18.8%)	0.598
Lipid disorders, n (%)	9 (60.0%)	11 (68.8%)	0.611
Smoking never/past/current, n (%)	6 (40.0%)/6 (40.0%)/3 (20.0%)	8 (50.0%)/6 (37.5%)/2 (12.5%)	0.797
sBP (mmHg)	113.1 ± 17.3	116.4 ± 15.8	0.584
dBP (mmHg)	68.7 ± 9.5	62.2 ± 9.7	0.068
Heart rate (bpm)	71.1 ± 7.4	67.1 ± 10.2	0.219
LVEF (%)	57.1 ± 17.6	56.8 ± 21.7	0.960
**Laboratory data**			
Hb (g/dL)	13.3 ± 2.2	12.5 ± 1.4	0.237
Alb (mg/dL)	3.8 ± 0.4	3.7 ± 0.4	0.474
eGFR (mL/min/1.73 m2)	70.3 ± 17.8	68.9 ± 20.2	0.851
AST (mg/dL)	20.6 ± 6.9	24.3 ± 9.0	0.218
ALT (mg/dL)	22.0 ± 8.6	29.9 ± 17.9	0.143
UA (mg/dL)	6.5 ± 2.2	6.4 ± 1.5	0.954
LDL (mg/dL)	87.9 ± 33.1	86.6 ± 25.4	0.907
HbA1c (%)	6.2 ± 1.4	6.1 ± 0.6	0.769
BNP (pg/dL)	65.7 [11.4–227.0] (n = 10)	54.9 [8.1–323.2] (n = 11)	0.526
NT-proBNP (pg/dL)	560.0 [56.7–850.0] (n = 3)	258.0 [8.4–3889.0] (n = 9)	0.340
**Medications, n (%)**			
Beta blockers	10 (66.7%)	10 (62.5%)	0.809
ACE-i/ARB	7 (46.7%)	8 (50.0%)	0.853
ARNI	1 (6.7%)	2 (12.5%)	0.583
Ca blocker	6 (40.0%)	8 (50.0%)	0.576
Loop diuretics	5 (33.3%)	7 (43.8%)	0.552
SGLT2i	2 (13.3%)	3 (18.8%)	0.682
**Exercise tolerance**			
Peak VO_2_ (mL/min/kg)	14.8 ± 4.7 (n = 10)	17.2 ± 5.1 (n = 11)	0.269
6MWT distance (m)	376.3 ± 102.7 (n = 6)	372.0 ± 68.5 (n = 5)	0.935
**SF-8**			
PCS	45.3 ± 6.4	45.5 ± 9.5	0.965
MCS	47.5 ± 8.5	47.3 ± 7.5	0.954
Physical Functioning	44.7 [44.7,54.3]	44.7 [44.7,54.3]	0.252
Role Physical	43.6 [43.6,54.9]	47.8 [43.6,54.9]	0.829
Bodily Pain	51.3 [45.0,59.1]	52.1 [37.1,59.1]	0.792
General Health	43.0 [43.0,52.7]	43.0 [36.4,60.0]	0.754
Vitality	47.4 [41.1,55.5]	47.4 [42.7,53.5]	0.967
Social Functioning	47.3 [33.1,55.2]	55.2 [41.4,55.2]	0.522
Role Emotional	49.0 [43.7,55.2]	55.2 [36.5,55.2]	0.9.9
Mental Health	51.2 [38.3,56.7]	45.1 [40.0,56.7]	0.586
Number of RCR times	26.9 ± 8.8	21.9 ± 8.3	0.133
Days of RCR	80.0 ± 20.5	77.9 ± 18.9	0.801
Days from admission to RCR	40.5 ± 22.7	33.1 ± 17.6	0.363

Abbreviations: ACE-i; angiotensin converting enzyme inhibitor, Alb; albumin, ARB; angiotensin II receptor blocker, ARNI; Angiotensin receptor/neprilysin inhibitor, ALT; alanine aminotransferase, AST; aspartate aminotransferase, BMI; body mass index, BNP; B-type natriuretic peptide, CR; cardiac rehabilitation, dBP; diastolic blood pressure, eGFR; estimated glomerular filtration rate, Hb; hemoglobin, LDL; low density lipoprotein, LVEF; left ventricular ejection fraction, MCS; Mental health component summary score, NT-proBNP; N-terminal pro-brain natriuretic peptide, PCS; physical health component summary score, RCR; remote cardiac rehabilitation, sBP; systolic blood pressure, SF-8; Short-Form Health Survey-8, SGLT2i; sodium-glucose cotransporter-2 inhibitor, UA; uric acid, VO_2_; oxygen consumption, 6MWT; 6-minute walk test.

**Table 2:** HRQOL measured by SF-8 before and after 2–3 months of RCR: 8 subscales in all populations or I and NI groups. The data are presented as “median (quadrant)”. We compared each score of HRQOL item between baseline and follow-up in all, I or NI group, respectively. * represents statistically significant.

	All			I Group N = 15			NI Group N = 16		
	Baseline	Follow-Up	*p*-Value	Baseline	Follow-Up	*p*-Value	Baseline	Follow-Up	*p*-Value
**Physical Functioning**	44.7 [44.7,54.2]	54.3 [48.7,54.3]	<0.001 *	44.7 [44.7,54.3]	54.3 [48.7,54.3]	0.002 *	44.7 [44.7,54.3]	54.3 [50.1,54.3]	0.002 *
**Role Physical**	47.8 [43.6,54.9]	54.9 [54.9,54.9]	0.001 *	43.6 [43.6,54.9]	54.9 [54.9,54.9]	0.003 *	47.8 [43.6,54.9]	54.9 [49.6,54.9]	0.078
**Bodily Pain**	51.3 [37.1,59.1]	59.1 [51.3,59.1]	0.027 *	51.3 [45.0,59.1]	59.1 [51.3,59.1]	0.168	52.1 [37.1,59.1]	59.1 [46.6,59.1]	0.094
**General Health**	43.0 [43.0,52.7]	52.7 [52.7,60.0]	<0.001 *	43.0 [41.1,55.5]	52.7 [52.7,60.0]	<0.001 *	43.0 [43.0,52.7]	52.7 [52.7,60.0]	0.003 *
**Vitality**	47.4 [41.1,55.4]	55.5 [47.4,55.5]	<0.001 *	47.4 [41.1,55.2]	55.2 [47.3,55.2]	0.001 *	47.4 [42.7,53.5]	55.5 [47.4,55.5]	0.025 *
**Social Functioning**	47.3 [41.4,55.2]	55.2 [47.3,55.2]	0.004 *	47.3 [33.1,55.2]	55.2 [49.0,55.2]	0.016 *	55.2 [41.4,55.2]	55.2 [49.2,55.2]	0.096
**Role Emotional**	55.2 [43.7,55.2]	55.2 [49.0,55.2]	0.015 *	49.0 [43.7,55.2]	56.7 [51.2,56.7]	0.015 *	55.2 [36.5,55.2]	55.2 [49.0,55.2]	0.102
**Mental Health**	45.1 [38.3,56.7]	56.7 [51.2,56.7]	0.003 *	51.2 [38.3,55.5]	55.5 [55.5,58.1]	0.029 *	45.1 [40.0,56.7]	56.7 [46.7,56.7]	0.047 *

Abbreviations: HRQOL; health related quality of life, I; exercise tolerance improvement, NI; non-improvement of exercise tolerance, PCS; physical health component summary score, RCR; remote cardiac rehabilitation, SF-8; Short-Form Health Survey-8.

**Table 3:** Correlation coefficients between changes in PCS and MCS and variables at baseline.

	Changes in PCS (N = 31)		Changes in MCS (N = 31)	
	r_s_	*p*-Value	r_s_	*p*-Value
**Age (years)**	0.19	0.301	0.17	0.359
**BMI (kg/m^2^)**	0.01	0.997	−0.06	0.757
**LVEF (Teichholz) (%) (N = 29)**	−0.51	0.792	0.39	0.036
**Peak VO_2_ (mL/min/kg) (N = 21)**	−0.11	0.626	−0.11	0.626
**Number of RCR times**	0.35	0.071	0.09	0.637
**Days of RCR**	0.04	0.861	−0.04	0.857
**Days from admission to RCR**	−0.17	0.388	−0.51	0.007
**Changes in peak VO_2_ (N = 20)**	−0.19	0.416	−0.04	0.870

Abbreviations: BMI; body mass index, LVEF; left ventricular ejection fraction, MCS; mental health component summary score, PCS; physical health component summary score, RCR; remote cardiac rehabilitation, VO_2_; oxygen consumption.

## Data Availability

The data that support the findings of this study are available on request from the corresponding author. The data are not publicly available due to privacy or ethical restrictions.

## Supplementary Materials

The following supporting information can be downloaded at: https://www.mdpi.com/article/10.3390/jcm14103265/s1, Supplemental Material File S1, Sensitivity analysis using different thresholds in the improvement of exercise tolerance.; Supplemental Material File S2, RecRCR investigators.

## Abbreviations

The following abbreviations are used in this manuscript:

ACEi	angiotensin converting enzyme inhibitor
Alb	albumin
ARB	angiotensin II receptor blocker
ARNI	angiotensin receptor/neprilysin inhibitor
ALT	alanine aminotransferase
AST	aspartate aminotransferase
BMI	body mass index
BNP	B-type natriuretic peptide
CPET	cardiopulmonary exercise testing
CR	cardiac rehabilitation
CVD	cardiovascular diseases
dBP	diastolic blood pressure
eGFR	estimated glomerular filtration rate
Hb	hemoglobin
HRQOL	health related quality of life
LDL	low density lipoprotein
LVEF	left ventricular ejection fraction
MCS	mental health component summary score
NT-proBNP	N-terminal pro-brain natriuretic peptide
PCS	physical health component summary score
QOL	quality of life
RCR	remote cardiac rehabilitation
sBP	systolic blood pressure
SF-8	Short-Form Health Survey-8
SGLT2i	sodium-glucose cotransporter-2 inhibitor
UA	uric acid
VO_2_	oxygen consumption
6MWT	6-minute walk test
