# Supplementary material for: Relationship Between Health-Related Quality of Life and Exercise Tolerance Improvement in Remote Cardiac Rehabilitation: Sub-Analysis of RecRCR Study
Source: J Clin Med. 2025 May 8;14(10):3265. doi: 10.3390/jcm14103265 (PMC12112100; doi:10.3390/jcm14103265)
Supplement: Supplementary file 1 [file jcm-14-03265-s001.zip › Supplementary materials 1 0417.pdf]

Supplementary materials 1

Table S1

Sensitivity analysis

When patients were divided into two groups at 5% of exercise capacity improvement, PCS and MCS changed during RCR as follows.

	I group (improvement > 5%)	NI group (improvement < 5%)
	baseline→follow-up	baseline→follow-up
N	22	9
PCS	44.9±8.1→51.8±4.4 (p=0.0006)	49.3±8.3→54.3±2.1 (p=0.021)
MCS	46.4±8.2→50.3±6.2 (p=0.013)	50.1±6.8→52.9±3.0 (p=0.23)

We also performed an analysis using only the improvement of peak VO2 in CPET.

	I group<br>improvement > 10% in peak VO2	NI group<br>improvement < 10% in peak VO2
	baseline→follow-up	baseline→follow-up
N	8	11
PCS	46.9±6.2→52.4±4.7 (p=0.0006)	45.3±9.4→51.7±4.8 (p=0.021)
MCS	45.1±7.4 →50.1±4.8 (p=0.013)	47.6±8.3→48.8±7.4 (p=0.22)

I; improvement, NI; no improvement, PCS; physical component scores, MCS; mental component scores, CPET; cardiopulmonary exercise test
