# Supplementary material for: Relationship Between Health-Related Quality of Life and Exercise Tolerance Improvement in Remote Cardiac Rehabilitation: Sub-Analysis of RecRCR Study
Source: J Clin Med. 2025 May 8;14(10):3265. doi: 10.3390/jcm14103265 (PMC12112100; doi:10.3390/jcm14103265)
Supplement: Supplementary file 1 [file jcm-14-03265-s001.zip › Supplementary materials 2 0417.pdf]

Supplementary Material 2 : RecRCR investigators

Saiseikai Futsukaichi Hospital: Hirofumi Yamamichi, Yuhiro Otsuka

Dokkyo Medical University Nikko Medical Center: Yuma Tamura, Masato Terashima

Nijigaoka Hospital: Mari Hamada, Akari Noda-Okumura

Moriyama Memorial Hospital: Yuhei Takahara, Atsushi Ichimura

Fukuoka Sanno Hospital: Toshie Tanaka, Hiroyuki Osato

Rokko Island Konan Hospital: Kazuma Hirose, Yoshihiro Shirota

Fukuoka University School of Medicine: Yasunori Suemastu, Kanta Fujimi

Shonan Hospital: Rie Takahashi, Masami Miyazato

Asahikawa Medical University Hospital: Tetsuo Oota, Takanori Ro

Kameda Medical Center: Yukio Ino, Shingo Muranaga

Kagawa University: Eriko Nasu, Masahiko Mizobuchi

Yamagata University School of Medicine: Takanori Arimoto, Masafumi Watanabe

Kanazawa University Hospital: Atsuhiro Iguchi, Yusuke Mizuno

Nihonkai General Hospital: Miyuki Yokoyama, Go Honma

Iwate Prefectural Central Hospital: Atsuhiro Iguchi, Yusuke Mizuno

Association of Healthcare corporation Kyufukukai Sekino Hospital: Hitomi Koshi

Juntendo University Graduate School of Medicine: Tetsuya Takahashi, Masakazu Saitoh

Nagoya University Graduate School of Medicine: Kiyonori Kobayashi, Toyoaki Murohara

Jichi Medical University Saitama Medical Center: Hideo Fujita, Hisataka, Maki

Steel Memorial Yawata Hospital: Chizuko Ikenaga, Tetsuro Nagata

Kurume University School of Medicine: Yoshihiro Fukumoto, Ken-Ichiro Sasaki

Shinshu University School of Medicine: Satoko Higuchi, Shuhei Yamamoto

Hiraka General Hospital: Satoru Takeda, Kenichi Sato

Konan Medical Center: Aya Fujiwara

Kansai Medical University: Takumi Miyauchi, Nana Takao

Gunma Prefectural Cardiovascular Center: Katsumi Hirai, Hiroko Kazama

Kofu Kyoritsu Hospital: Yasumichi Maejima, Kiriko Hagihara

Hyogo Prefectural Amagasaki General Medical Center: Kozo Hotta, Yukihito Sato

Hokkaido Cardiovascular Hospital: Ryota Sumiyoshi, Nao Hashida

Kyoto Prefectural University of Medicine: Shiho Yamabata, Fumika Kita
